# Production of Conductive PEDOT-Coated PVA-GO Composite Nanofibers

**DOI:** 10.1186/s11671-017-1888-0

**Published:** 2017-02-13

**Authors:** Nur Afifah Zubair, Norizah Abdul Rahman, Hong Ngee Lim, Yusran Sulaiman

**Affiliations:** 1 Department of Chemistry, Faculty of Science, Universiti Putra Malaysia, 43400 Serdang, Selangor Malaysia; 2 Functional Device Laboratory, Institute of Advanced Technology, Universiti Putra Malaysia, 43400 Serdang, Selangor Malaysia

**Keywords:** Poly(3,4-ethylenedioxythiophene), Electrospinning, Electrodeposition

## Abstract

Electrically conductive nanofiber is well known as an excellent nanostructured material for its outstanding performances. In this work, poly(3,4-ethylenedioxythiophene) (PEDOT)-coated polyvinyl alcohol-graphene oxide (PVA-GO)-conducting nanofibers were fabricated via a combined method using electrospinning and electropolymerization techniques. During electrospinning, the concentration of PVA-GO solution and the applied voltage were deliberately altered in order to determine the optimized electrospinning conditions. The optimized parameters obtained were 0.1 mg/mL of GO concentration with electrospinning voltage of 15 kV, which displayed smooth nanofibrous morphology and smaller diameter distribution. The electrospun PVA-GO nanofiber mats were further modified by coating with the conjugated polymer, PEDOT, using electropolymerization technique which is a facile approach for coating the nanofibers. SEM images of the obtained nanofibers indicated that cauliflower-like structures of PEDOT were successfully grown on the surface of the electrospun nanofibers during the potentiostatic mode of the electropolymerization process. The conductive nature of PEDOT coating strongly depends on the different electropolymerization parameters, resulting in good conductivity of PEDOT-coated nanofibers. The optimum electropolymerization of PEDOT was at a potential of 1.2 V in 5 min. The electrochemical measurements demonstrated that the fabricated PVA–GO/PEDOT composite nanofiber could enhance the current response and reduce the charge transfer resistance of the nanofiber.

## Background

Recently, nanofibers have been broadly investigated as high-performance materials due to their unique and remarkable properties which exhibit tremendous advantages. Nanofibers have attracted a great deal of attention because of their excellent characteristics including a high surface area to volume ratio, low specific mass, and extensive porosity [1] which make them appropriate for a diverse range of applications. Due to their fascinating properties, research on the fabrication of conductive polymer nanofibers has become a prominent research area and remains as one of the most important fields which attracted great attention. These nanofibers have been utilized extensively as electrochromic devices, chemical sensors [2], drug delivery [3], wound dressings [4], and photovoltaic devices [5]. In recent years, various attempts were made in the modification of nanofibers to improve their properties for specific applications [6, 7].

Previously, various techniques have been employed to obtain suitable polymer nanofibers for different purposes such as electrospinning, template synthesis, drawing, phase separation, and self-assembly [8]. At present, electrospinning is one of the most versatile and promising techniques for fabricating nanoscale fibers with relatively low cost, scalability, high productivity, and ability to produce long continuous fibers. More than 200 polymers have been successfully electrospun from a number of naturals and synthetic polymers, which characterized based on their potential applications [9]. The transformation of polymer solutions into nanofibers in the electrospinning process is affected by several parameters including the solution and processing condition [10].

Poly(3,4-ethylenedioxythiophene) (PEDOT) as one of the conductive polymers from polythiophene derivatives has been widely explored due to its excellent environmental and thermal stability, high-speed electron transfer, good mechanical strength, and relatively high optical transparency when in its electrically conductive state [11]. However, it is difficult to use intrinsically conducting polymers (ICPs) in the electrospinning process for nanofiber production due to its intrinsic high crystallinity [12], low solubility in general solvents [13], and too stiff to be electrospun by themselves [14]. Therefore, a combination of ICPs with another electrospinnable polymer is effectively considered to fabricate nanofibers but this may lead to the detriment of the electronic properties. Hence, despite declining its special characteristics, a facile method was studied to fabricate highly conductive nanofiber by coating electrospun nanofibers with conductive materials. This method is adjustable and essentially enhances the electrical properties of the electrospun nanofiber. Additionally, introducing conducting polymer onto the polymer nanofiber has a potential for producing electroactive nanomaterials. Current studies reported upon the deposition of conducting polymers on electrospun nanofiber structures, the template could potentially produce a promising class of nanomaterials with highly porous structure, large surface area to volume ratio, [15], and an ultrathin conducting layer [1], thus make such materials attractive for various applications. Therefore, electrochemical polymerization is practically more preferred for coating purpose compared to other techniques due to its controllable, easy to prepare, fast, and convenient [16] method to obtain high conductivity of PEDOT layer on the electrospun nanofibers.

Another important approach in producing polymer-based composites is the incorporation of carbon-based materials such as carbon nanotubes (CNT) and graphene derivatives into the nanofibers. The complete dispersion of nanofiller represents another challenge that can be countered by specific material, in order to increase their affinity for the polymer matrix in an appropriate solvent. This issue can be overcome by selecting a kind of effective nanofiller to be encapsulated into an electrospun nanofiber matrix. In this study, graphene oxide (GO) is appointed as the best candidate for nanofillers to be reinforced with a hydrophilic polymer such as polyvinyl alcohol (PVA) due to its abundance of hydrophilic groups on its surface. Additionally, PVA is considered as a commonly used electrospinnable polymer due to its processibility, good biodegradability, non-toxicity, and good mechanical properties [17]. Therefore, the high dispersibility of GO in water makes it the best material as nanofiller to be incorporated with PVA matrices. Moreover, GO as one of the most important graphene derivatives has attracted tremendous interest because of their unique properties which are high surface area and good electrical conductivity. As reported by Rose et al. [18], polymers will exhibit excellent properties after being reinforced with nanofillers, which contribute to the high performances of materials. The incorporation of nanofillers into electrospun nanofibers improves their properties to a certain extent. In addition, the special criteria of nanofibers which make them commercially important are their role in enhancing the electroactive nature, high surface area, and high flexibility in surface functionalities.

In this study, conductive nanofibers were fabricated by a dual process using electrospinning and electropolymerization techniques. An attempt has been made to optimize the conditions for electrospinning and electropolymerization to obtain uniform and continuous nanoscale fibers with good electrical conductivity. During the electrospinning, optimization of some parameters was carried out to obtain the smallest diameter of PVA and PVA-GO nanofibrous structures. Finally, the effect of the electropolymerization potential and time of PEDOT onto the electrospun nanofibers was studied by electrochemical measurements.

## Methods

### Materials

Polyvinyl alcohol (PVA) (Mw 89,000–98,000, 99+% hydrolyzed), 3,4-ethylenedioxythiophene (EDOT, 99%), lithium perchlorate (LiClO_4_, 95.0%) were purchased from Sigma Aldrich. Acetonitrile (CH_3_CN, 99%) was obtained from J.T.Baker. Potassium ferricyanide (K_3_[Fe(CN)_6_]), potassium ferrocyanide (K_4_[Fe(CN)_6_]), and KCl were obtained from BDH Analar. All reagents in this experiment were in analytical grade and used as received without further purification. The indium tin oxide (ITO) glass substrate was purchased from Xin Yan Technology Ltd. The ITO glass substrate was cleaned by sonication in acetone, ethanol, and deionized water (DI) sequentially for 15 min each. DI water (resistivity ~18.2 MΩ) was used throughout the experiments.

### Preparation of GO Suspension

GO suspension was successfully prepared using modified Hummer’s method [19]. Graphite powder was mixed with concentrated H_2_SO_4_ and continuously stirred in an ice bath for 10 min. Then, KMnO_4_ was slowly added into the above solution with vigorous stirring for 6 h and the temperature of the mixture was kept below 20 °C. The mixture was then reacted for 2 h before being transferred to a 40 °C water bath with vigorously stirred. Next, the temperature of the solution was adjusted to a constant 90 °C for 60 min while water was added continuously. The mixture was stirred overnight for complete oxidation of the graphite. During the oxidation, the color of the suspension changed from dark purplish green to dark brown. Distilled water was added to the suspension followed by a few drops of H_2_O_2_ to stop the oxidation process. The resultant GO suspension was centrifuged at 10 000 rpm and washed with 1.0 M HCl in aqueous solution to remove the unexfoliated particles. Then, the centrifuged solution was rinsed with deionized water until the pH of the solution became neutral. The successful formation of GO was verified by Fourier-transform infrared spectroscopy (FTIR) [20], Raman spectroscopy, and X-ray diffraction [21].

### Preparation of PVA and PVA-GO Nanofibers by Electrospinning

PVA solution was prepared by dissolving PVA powder in DI water with a weight percentage of 10 *w*/*v*%. The solution was gently stirred for 2 h and the temperature was maintained at 80 °C–90 °C until all the PVA powder was completely dissolved. For the preparation of PVA-GO solutions, different amount of GO suspension was added to the aqueous PVA solution to form various GO concentrations (0.05, 0.1, 0.3, and 0.5 mg/mL). The mixture was stirred further for 1 h in order to make sure the GO suspension was completely dispersed in the solution. The mixture was then sonicated at room temperature for 15 min to promote dispersion and form a homogeneous mixture before being subjected to the electrospinning process.

Nanofiber Electrospinning Machine was used for the preparation of nanofibers. The polymer solution was loaded into a glass syringe (5 mL), and a flexible tube was used to connect the syringe to a 15G stainless-steel needle with an inner diameter of 14 mm. The needle was connected to a high-voltage power supply which generated DC voltages in the range of 10–25 kV during electrospinning. The setup was equipped with a syringe pump to control the flow rate of the solution which was fixed precisely to 1.2 mL/h for this study. Nanofibers were collected on the ITO glass (1 cm^2^) which was attached to the grounded metal collector covered with aluminum foil. The ITO glass was stacked on the collector whose surface was in the same plane of the collector by double-sided tape. The distance from the needle to the collector was 15 cm and the nanofiber production time was set to 15 min. The electrospinning was performed in a closed chamber at room temperature, and the resulting nanofibers were kept dried in a desiccator for few days to ensure complete drying of the sample prior polymerization of PEDOT. In this study, the optimization of the voltage applied during electrospinning was carried out.

### Electropolymerization of PEDOT onto PVA-GO Nanofibers

**Fig. 1:**
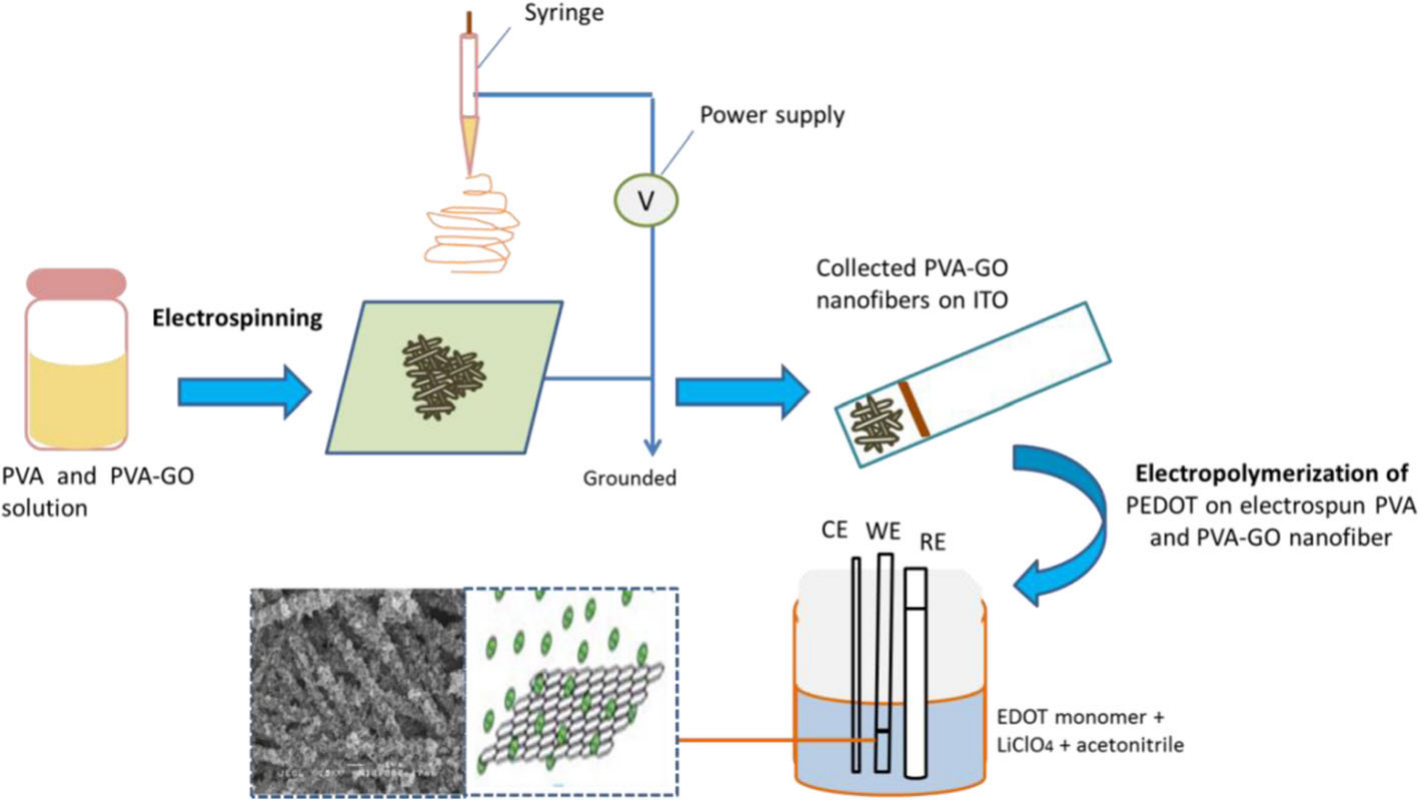
Schematic diagram illustrating the steps involved in the fabrication of PVAGO/PEDOT nanofibers prepared by combining electrospinning and electropolymerization methods

Electropolymerization of PEDOT onto collected nanofibers was performed in non-aqueous medium containing 0.01 M EDOT and 0.1 M LiClO_4_ as supporting electrolyte in acetonitrile. One compartment of electrochemical cell consists of three electrode systems was used for electropolymerization. The ITO glass substrates coated with PVA-GO nanofibers were used as working electrode with fixed deposition area (1 cm^2^), a platinum wire as a counter electrode and silver wire coated with silver chloride as a pseudo-reference electrode. Preliminary experiment using cyclic voltammetry was performed in order to determine the onset potential for the polymerization of PEDOT. Consequently, the electropolymerization was carried out by a chronoamperometric method with at different applied potential (1.0, 1.2, and 1.5 V) with a scan rate of 0.1 V/s. These samples will hereafter be called as PVA-GO/PEDOT1.0V, PVA-GO/PEDOT1.2V, and PVA-GO/PEDOT1.5V. The samples prepared without the presence of PVA-GO nanofibers will be called as PEDOT1.0V, PEDOT1.2V, and PEDOT1.5V. The effect of electropolymerization time on the PEDOT-coated nanofiber was studied by varying the time from 1 to 15 min at a fixed potential (1.2 V). Hereafter, the sample prepared at different electropolymerization time will be denoted as PVA-GO/PEDOT1m, PVA-GO/PEDOT5m, PVA-GO/PEDOT10m, and PVA-GO/PEDOT15m. PEDOT was also electropolymerized on ITO glass without PVA-GO nanofibers as a comparison and will be denoted as PEDOT1m, PEDOT5m, PEDOT10m, and PEDOT15m. Figure 1 shows the schematic of the experimental process for the preparation of nanofibers by the combination of electrospinning and electropolymerization techniques.

### Characterization

The morphology of the nanofibers on the surface of indium tin oxide-coated glass was examined by JOEL JSM-6400 scanning electron microscope (SEM). The samples were mounted on aluminum stubs using double-sided carbon-coated tape and were vacuumed and gold coated for 10 min prior to analysis by using sputter coater. The analysis was carried out at accelerating voltage of 10–15 k. A computer-controlled potentiostat/galvanostat (Metrohm Autolab PGSTAT204 operated with NOVA software, version 1.10) was used for cyclic voltammetry (CV) and electrochemical impedance spectroscopy (EIS) measurements. The measurements were carried out in a standard three-electrode setup with a platinum coil as the counter electrode and Ag/AgCl used as the reference electrode. The obtained nanofiber on the ITO glass was used as the working electrode. In this study, CV measurements were performed in 10 mM [Fe(CN)_6_]^3−^ as a redox probe containing 0.1 M KCl as supporting electrolyte. The CV potential range was scanned from −0.2 to 0.6 V with varied scan rate from 2 to 100 mV/s at room temperature. Electrochemical impedance spectroscopy (EIS) was used to study the electrochemical and surface reactions of electrodes. This technique gives information about the interfacial properties of PEDOT-coated nanofibers. The measurements were carried out to in solution consists of equimolar 5 mM [Fe(CN)_6_]^3−/4−^ redox system containing 0.1 M KCl using a frequency range between 0.01 and 10 kHz at open circuit potential (OCP), by using a sinusoidal excitation signal (single sine) with an excitation amplitude of 10 mV.

## Results and Discussion

### Preparation of Nanofibers by Electrospinning

#### Effect of Different Applied Voltage

**Fig. 2:**
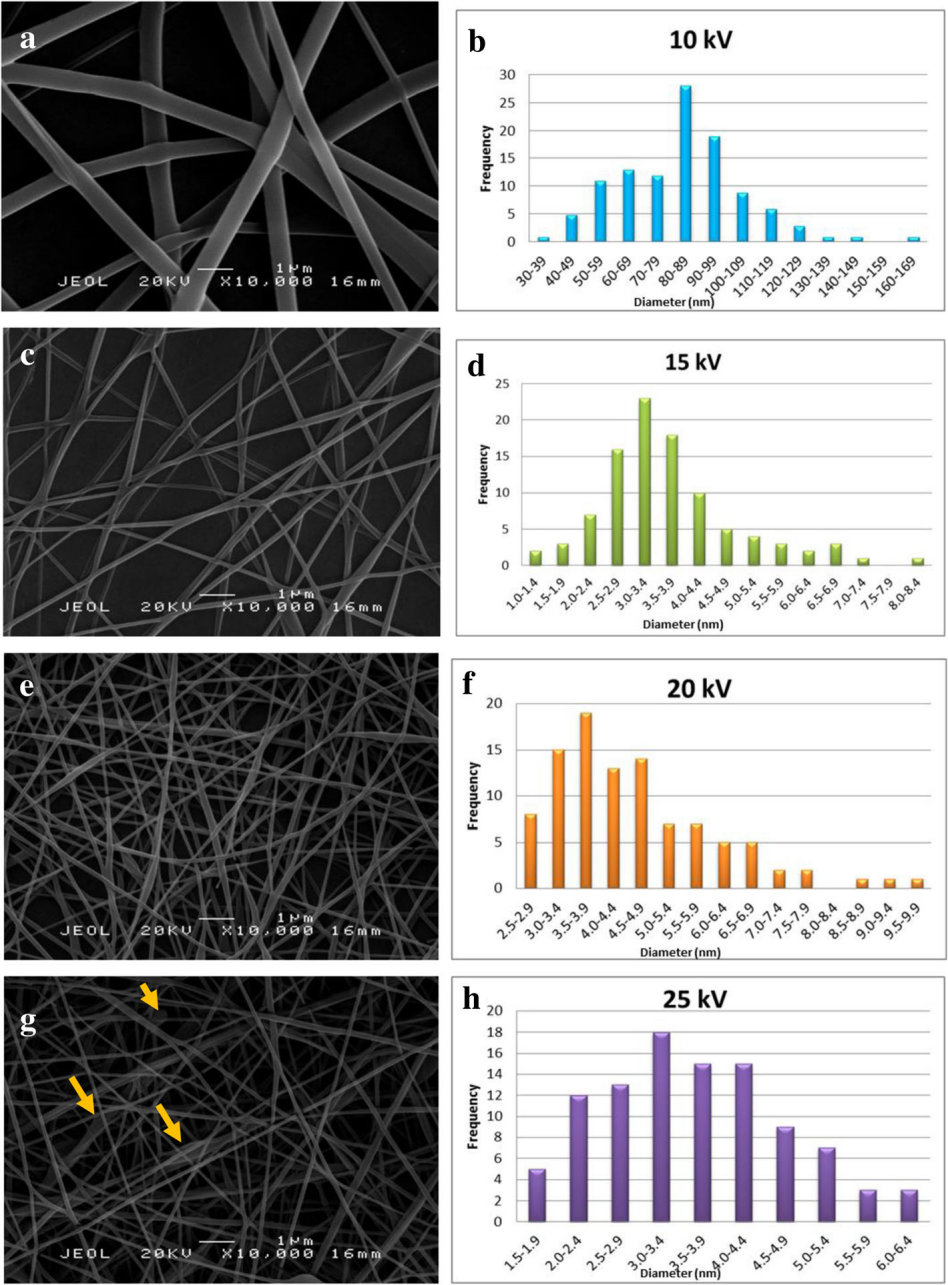
SEM micrographs and the diameter distribution of 10 *w*/*v*% PVA nanofibers at **a**, **b** 10 kV, **c**, **d** 15 kV, **e**, **f** 20 kV, and **g**, **h** 25 kV applied voltage with a spinning distance of 15 cm at 10,000× magnification

Four different voltages were applied (10, 15, 20, and 25 kV) to prepare PVA nanofibers while the other parameters were kept constant and the SEM micrographs are displayed in Fig. 2. At low applied voltage (10 kV), the jet formation is insufficiently stretched due to the lower electric field and this effect leads to a larger diameter range of nanofibers as shown in Fig. 2a. The larger in diameter reflects that the voltage threshold must be exceeded for the formation of stable solution jet. When a higher voltage was applied, the ejection of polymer solution increases and this facilitates the greater stretching of the solution as well as a stronger electric field [22]. The SEM images revealed that an increase in the voltage from 15 to 25 kV and the average diameter of nanofiber increases from 250 ± 184 to 300 ± 126 nm. Increasing the applied voltage enhances the magnitude of the electric field as well as the electrostatic repulsive force which ultimately results in the thinner liquid jet. As a result, the spinning jet would get greater tensile stress and acceleration, which facilitates the formation of thinner nanofibers [23]. The polymer solution was removed from the capillary tip faster as the jet ejected from Taylor cone.

However, increasing the spinning voltage would contribute to the accumulation of charged and draw more solution out of the capillary tip which resulted in the difficulty of jet elongation [24]. This effect leads to the formation of spindle-like beads as can be observed in Fig. 2g (pointed by arrow). The inconsistency of the nanofibers diameter could probably be attributed to a competition between electrostatic force and polymer solution removed from a capillary [23]. The SEM images show that at 15 kV applied voltage, smooth and uniform nanofibers without beads was obtained; hence, it will be used throughout this work to study the effect of GO concentration in the polymer solution.

#### Effect of Different GO Concentrations

**Fig. 3:**
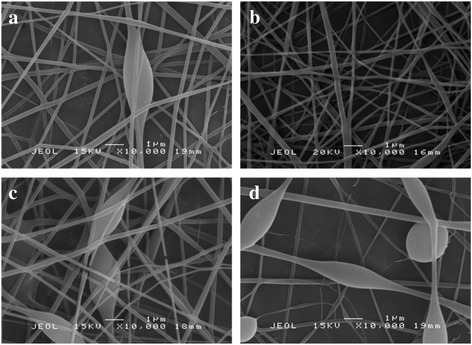
SEM micrographs of PVA-GO with **a** 0.05 mg/mL, **b** 0.1 mg/mL, **c** 0.3 mg/mL, and **d** 0.5 mg/mL of GO loading with 15 kV applied voltage and spinning distance of 15 cm at 10,000× magnification

The optimized parameters to produce PVA nanofibers were used to fabricate PVA-GO nanofibers. GO acts as a nanofiller to enhance the properties of the electrospun nanofibers. As suggested by Jiang et al. [25], the incorporation of polymer matrices with GO can provide more electrochemical activity sites within the nanofiber which exhibits excellent reinforcement effects to the nanocomposite materials. Figure 3 shows the SEM micrographs of PVA-GO nanofibers obtained using different concentrations of GO in PVA solution (0.05, 0.1, 0.3, and 0.5 mg/mL GO). It can be observed that there is no significant effect on the diameter of electrospun nanofibers but some beaded structures are formed as the concentration of GO is increased. As expected, a small amount of GO remarkably altered the solution viscosity of PVA solution, which may lead to instability of the liquid jet during the electrospinning process [26]. Apparently, it should be at the optimum condition to obtain smooth and continuous fibrous morphology since the properties of the GO sheets are hygroscopic in nature [27] which brings to poor long-term stability due to GO encapsulation.

As can be clearly seen in Fig. 3a, at low GO content (0.05 mg/mL), droplets are formed due to the low viscosity of the solution. Since the viscoelastic force is not sufficient to overcome the repulsive forces of charge, the charged jet is fragmented into discrete droplets before reaching the collector. However, at 0.1 mg/mL GO, continuous and smooth nanofibers with fewer beads and droplets were produced with an average diameter of 34.25 ± 12.61 nm. As the GO content is increased to 0.3 mg/mL, a combination of nanofibers and droplets is formed. A further increase in the GO concentration to 0.5 mg/mL has resulted in a large number of beads. The nanofibers are seen to be discontinuous and the shape becomes more spherical. The flow of the polymer solution through the capillary is disrupted caused the polymer jet to break up into droplets. It is deduced that the aggregation of GO sheets tends to occur when the content is above 0.1 mg/mL which causes by poor spin ability of the solution. This phenomenon can be explained as the content of GO increases and the dispersibility of GO sheets in the polymer matrices becomes poor [28]. The solution tends to aggregate during electrospinning procedure, which leads to an increase in the number of bead defect structures. Beadless nanofibers are preferred since they provide a higher surface area to volume ratio, which is the most desirable property for improving the electrochemical performances [29]. Moreover, the presence of beaded fibers could hinder the nanostructural characteristic and the effective surface area.

### Electropolymerization of PEDOT onto PVA-GO Nanofiber

The coating of nanofibers with PEDOT could be an efficient alternative to produce highly conductive nanofibers with good electrochemical performances. For coating purposes, an effective technique namely chronoamperometric was introduced, which can produce PEDOT polymer layer on PVA and PVA-GO nanofibers with high conductivity values. Therefore, the cyclic voltammetry measurement was performed to determine the onset oxidation potential and the suitable potential range for the electropolymerization of PEDOT on the electrospun nanofibers. The electropolymerization potential is important in the preparation of the polymer as it could affect the growth of the polymer process and might cause some changes in the structure of the polymer film. Therefore, it is crucial to choose the appropriately applied potential for the electropolymerization process.

**Fig. 4:**
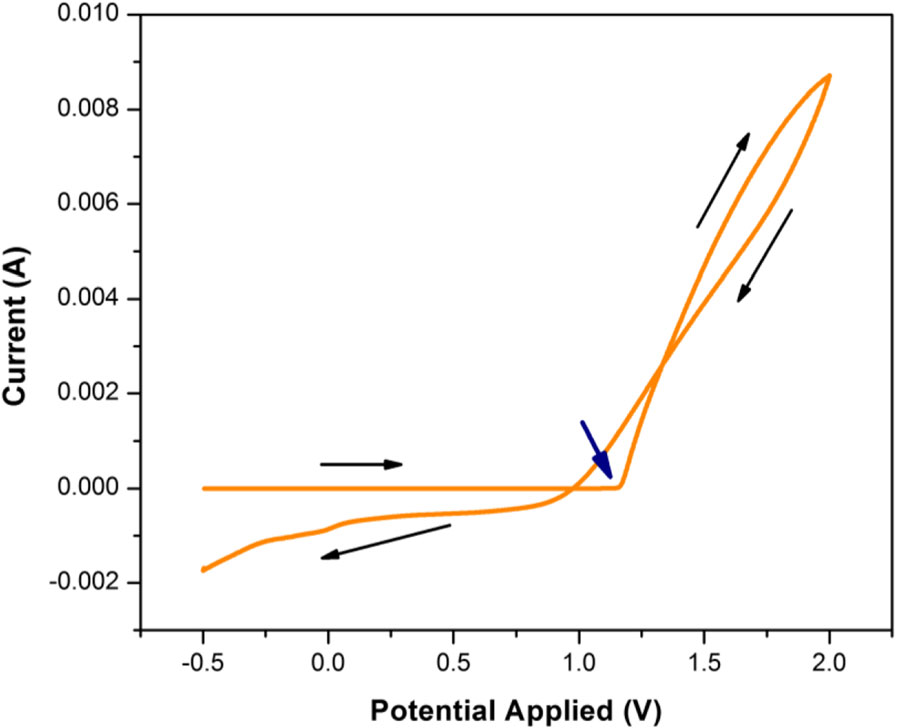
CV for electropolymerization of PEDOT from 0.01 M EDOT and 0.1 M LiClO4 in acetonitrile with a scan rate of 100 mV/s

Figure 4 shows the first cycle of CV for electropolymerization of PEDOT on ITO scanned between −0.5 and 2.0 V. The electropolymerization was performed from a solution containing 0.01 M EDOT and 0.1 M LiClO_4_ as supporting electrolyte in acetonitrile. The onset potential was determined from the intersection of the tangents drawn at the baseline current and the oxidation current slope in the CV [30]. It was found that the value of the onset potential (blue arrow) of PEDOT is approximately at 1.2 V. As a consequence, the electropolymerization of PEDOT was assigned at three different potentials (1.0. 1.2, 1.5 V) to study the difference of PEDOT-coating conditions.

A crossover was observed between the forward scan and reverse scan in the CV, called as “nucleation loop.” This phenomenon is attributed to the initial stage of nucleation process for conductive polymer film which corresponds to the theory of metal deposition [8]. The oxidation current (anodic) is observed at positive potentials higher than 1.2 up to 2.0 V. This feature is attributed to the beginning of oxidation for EDOT monomer to become polymer through a diffusion process. At this stage, generation of radical cation species, EDOT^•+^, occurs which initiates the polymerization process. The initiation step involves the anodic oxidation of EDOT monomers to radical cations which then dimerize and deprotonate. After the deprotonating step, the dimer is reoxidized and the process continues with the formation of oligomer radical cation species, which interact with other EDOT^•+^, forming the PEDOT polymer. The electrochemical polymerization of EDOT is described by Diaz’s mechanism [31]. Moreover, it was noticed that there is no reduction peak, indicating that the electropolymerization process of PEDOT is irreversible [32].

#### Effect of Different Electropolymerization Potentials

**Fig. 5:**
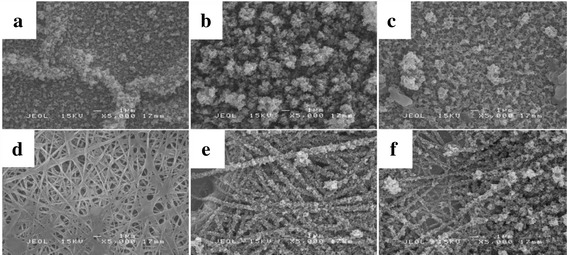
SEM micrographs of PEDOT electropolymerized on **a**–**c** ITO and **d**–**f** PVA-GO nanofibers at **a**, **d** 1.0, **b**, **e** 1.2, and **c**, **f** 1.5 V for 5 min

Figure 5 shows the SEM micrographs of PEDOT deposited on ITO and PVA-GO nanofibers for 5 min at different polymerization potentials. PEDOT1.0V (Fig. 5a) shows the growth of small globules with some areas is agglomerated and the globules grow larger with the increasing of potential to 1.2 V (Fig. 5b), resulting in the compact and densely packed structure with a thicker polymer film. However, as the potential of deposition is increased to 1.5 V, there is an increase in porosity of the PEDOT and a spongy nature of the surface (Fig. 5c). Similar morphology was observed by Zhang et al. [33] which reported loose spongy network is obtained for PEDOT prepared in an organic medium.

The deposition of PEDOT on PVA-GO nanofibers displays significant changes in the PEDOT morphologies. The surface of the PVA-GO/PEDOT1.0V nanofibers (Fig. 5d) displays spider web-like structure with a very thin layer of PEDOT. As the deposition potential increased to 1.2 V (Fig. 5e), PEDOT is homogeneously distributed on nanofibers which displays fibrous morphology, indicating that PEDOT is well coated on the surface of electrospun PVA-GO nanofiber without disrupted the fibril-like structure of nanofibers. Notably, increasing the deposition potential to 1.5 V, there is an increase in porosity of the samples which demonstrate that porosity increases by increasing the applied potential. However, at this condition, PEDOT is not uniformly distributed and the diameter of nanofibers becomes larger. Likewise, some larger globules have grown on a certain surface area of PVA-GO nanofibers as can be seen in Fig. 5f. This can be explained that the electric field at the electrode interface is less uniform when the applied voltage is too high, which results in the thicker deposition and rougher morphology of nanofibers [34].

#### Effect of Different Electropolymerization Times

**Fig. 6:**
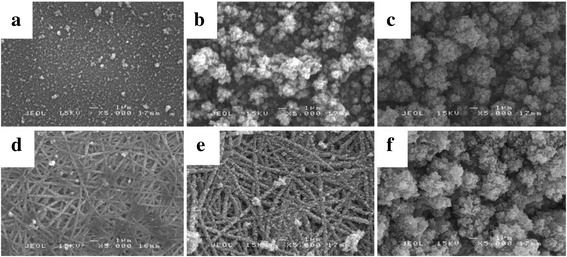
SEM micrographs of PEDOT electropolymerized on **a**–**c** ITO and **d**–**f** PVA-GO nanofibers for **a**, **d** 1, **b**, **e** 10, and **c**, **f** 15 min

Different electropolymerization time of PEDOT was employed to control the thickness and surface morphologies of nanofibers. The electropolymerization time was varied from 1 to 15 min to obtain a homogenous and highly conductive coating of PEDOT on the electrospun nanofibers. Figure 6 shows the SEM micrographs of nanofiber with different electropolymerization times of PEDOT on ITO and PVA-GO nanofibers at 1.2 V. It can be seen clearly that the uniformity and homogeneity were distinctly affected by time deposition. As depicted in Fig. 6a, the morphology of PEDOT1m displays small bulges scattered on the surface of ITO substrate. This might be due to the insufficient time for PEDOT to completely electropolymerized. As a result, the uncoated PVA-GO nanofiber surface is noticeable which leads to the void formation (Fig. 6d).

However, as the deposition time is extended to 5 min, there is a formation of the cauliflower-like structure of PEDOT nanoparticles (Fig. 6b). It was noted that the size of PEDOT particles increases with the longer electropolymerization time. This occurrence could be due to the larger number of PEDOT particles reaching the electrode and depositing on the substrate. As reported by Zhou et al. [34], the charge density is considered as a measure of the mass of deposited PEDOT particles. Therefore, the quantity of PEDOT deposited on the substrate increases with the increasing of the charge passed to the electrode.

A similar pattern was observed for PEDOT electrodeposited on the surface of PVA-GO nanofibers. As depicted in SEM images, the diameter of PVA-GO/PEDOT10m (Fig. 6e) nanofibers is larger than that of PVA-GO/PEDOT5m (Fig. 6e), indicating that PEDOT-coated nanofibers became thicker and more compact with the increasing deposition time. However, the adhesion of PEDOT on nanofiber was still in good condition until 10 min time deposition. As the growth of PEDOT on nanofiber is further extended to 15 min, the agglomeration and rougher surface of PVA-GO/PEDOT15m (Fig. 6f) were observed. The excess PEDOT particles started to combine and stack together to create a thicker layer of coating as the deposition time was prolonged, leading to the increment in the thickness and surface roughness. As a result, the PVA-GO nanofibers are fully covered with PEDOT particles; therefore, the fibrous morphology is not observable.

### Cyclic Voltammetry Measurement

In order to understand the electrochemical behavior of PEDOT deposited on nanofibers at different electropolymerization potentials, cyclic voltammetry measurements were swept between −0.2 and 0.6 V in 1.0 mM K_3_Fe(CN)_6_ containing 0.1 M KCl solution. The values of the peak current for the prepared nanofibers were measured by extrapolating the preceding baseline current.

**Fig. 7:**
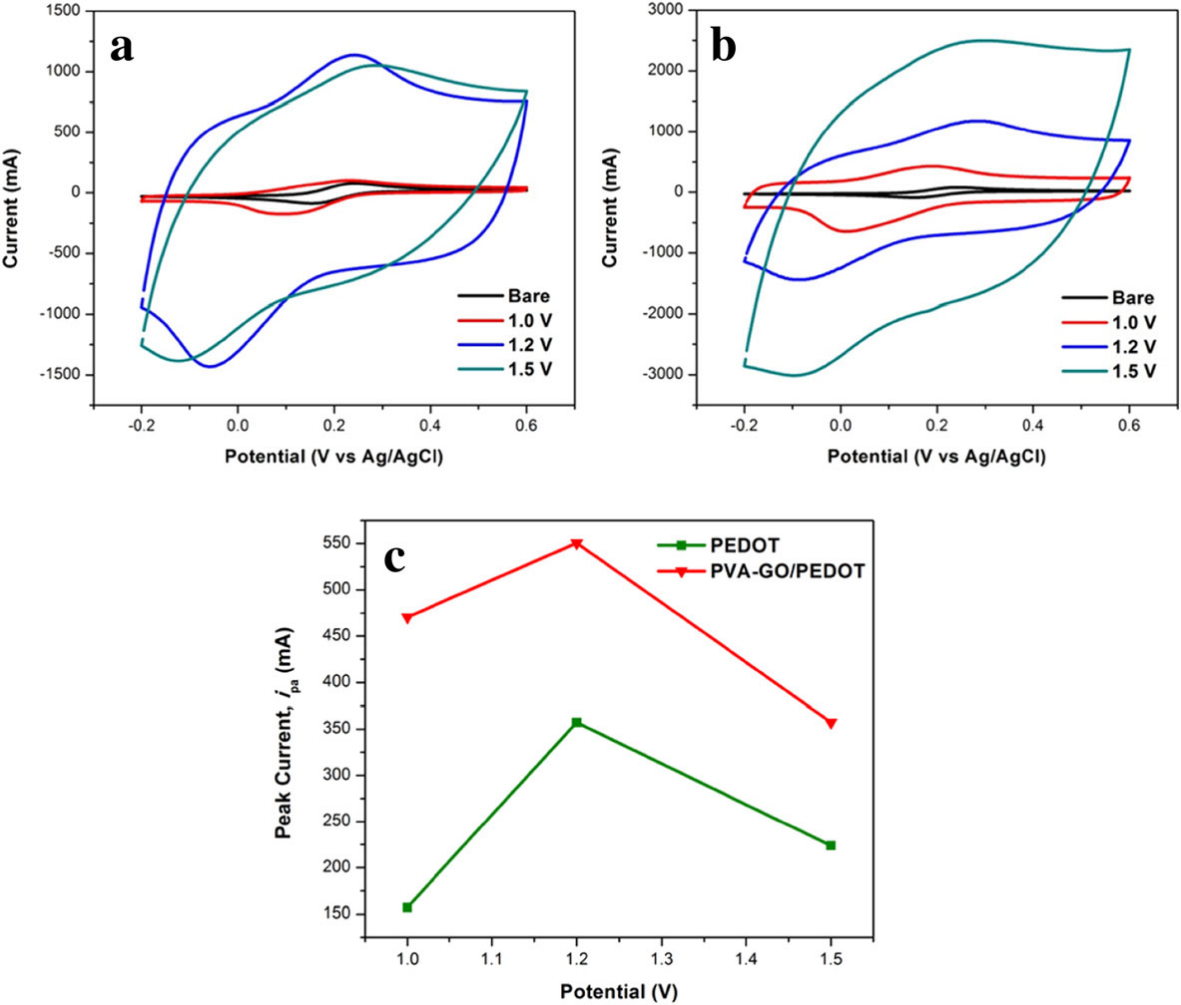
CVs of **a** PEDOT **b** PVA-GO/PEDOT prepared at different electropolymerization potentials of PEDOT in 1.0 mM K3Fe(CN)6 containing 0.1 M KCl solution. **c** Plot of anodic peak current at various electropolymerization potentials

As can be seen in Fig. 7a, b, the cyclic voltammogram of bare electrode displays the lowest current response. After the electrodeposition of PEDOT on bare ITO and PVA-GO nanofibers, the voltammetric response apparently improved, indicating PEDOT exhibits good electrochemical behavior [35]. However, the current response is dependent on the properties of PEDOT on the nanofiber. Therefore, attempts were made to study the relationship between the electropolymerization potential and the electrochemical properties of the conductive nanofibers obtained.

It was observed that when the electropolymerization potential of PEDOT on bare ITO is increased from 1.0 to 1.2 V, the peak current increases (Fig. 7c). A similar trend was observed for PEDOT electrodeposited on PVA-GO nanofibers. As can be seen in SEM images (Fig. 7a), the particles of PEDOT1.0 are rather small and inhomogeneous, which resulted in a low current response. However, PEDOT electrodeposited on PVA-GO nanofiber exhibits higher peak current compared to bare ITO (Fig. 7c), indicating PEDOT on PVA-GO nanofibers has larger active area due to the presence of a high surface area of nanofiber structure.

A drastic drop in the peak current is noticed as the electropolymerization potential is further increased to 1.5 V. This might be due to the structure of PEDOT becomes denser and the fibrous structures of the coated nanofibers become less pronounce as observed in the SEM images. Additionally, similar behavior has been reported that PEDOT electrodeposited at high potential (over 1.4 V) may undergo overoxidation which leads to degradation of PEDOT and decreases the conductivity of polymer film [36]. Therefore, the destruction of PEDOT-coated layer has partially blocked the movement of the redox active ions to the electrode surface. Hence, it could be explained that the electropolymerization potential of PEDOT affects the nucleation and growth process of the polymer on the PVA-GO nanofiber surface [37]. The electrodeposition potential of the polymer may affect the rate of monomer oxidation and the polymer chain formation, which demonstrates that at higher electrodeposition potential, the polymer chain becomes more packed, resulting in compact PEDOT layer.

In contrast, the nanofibrous structure of PVA-GO/PEDOT1.2V is seen clearly which certainly increases the active surface area of the nanofibers. The CV revealed that both anodic and cathodic peak currents are clearly defined compared to PVA-GO/PEDOT1.5V nanofibers (Fig. 7b). This phenomenon can also be observed for both PEDOT electrodeposited on bare ITO and PVA-GO nanofibers. Thus, it can be concluded that PEDOT1.5V causes the peak current suppressed due to the presence of a compact and dense structure of PEDOT. Thus, the ion transportation on the dense and compact structure of PEDOT film is limited and, consequently, increases the diffusion resistance between the electrode and electrolyte. Hence, the low current response observed is due to the excessive compactness of PEDOT coating on PVA-GO nanofiber. Therefore, 1.2 V was chosen as the electrodeposition potential for the next study.

**Fig. 8:**
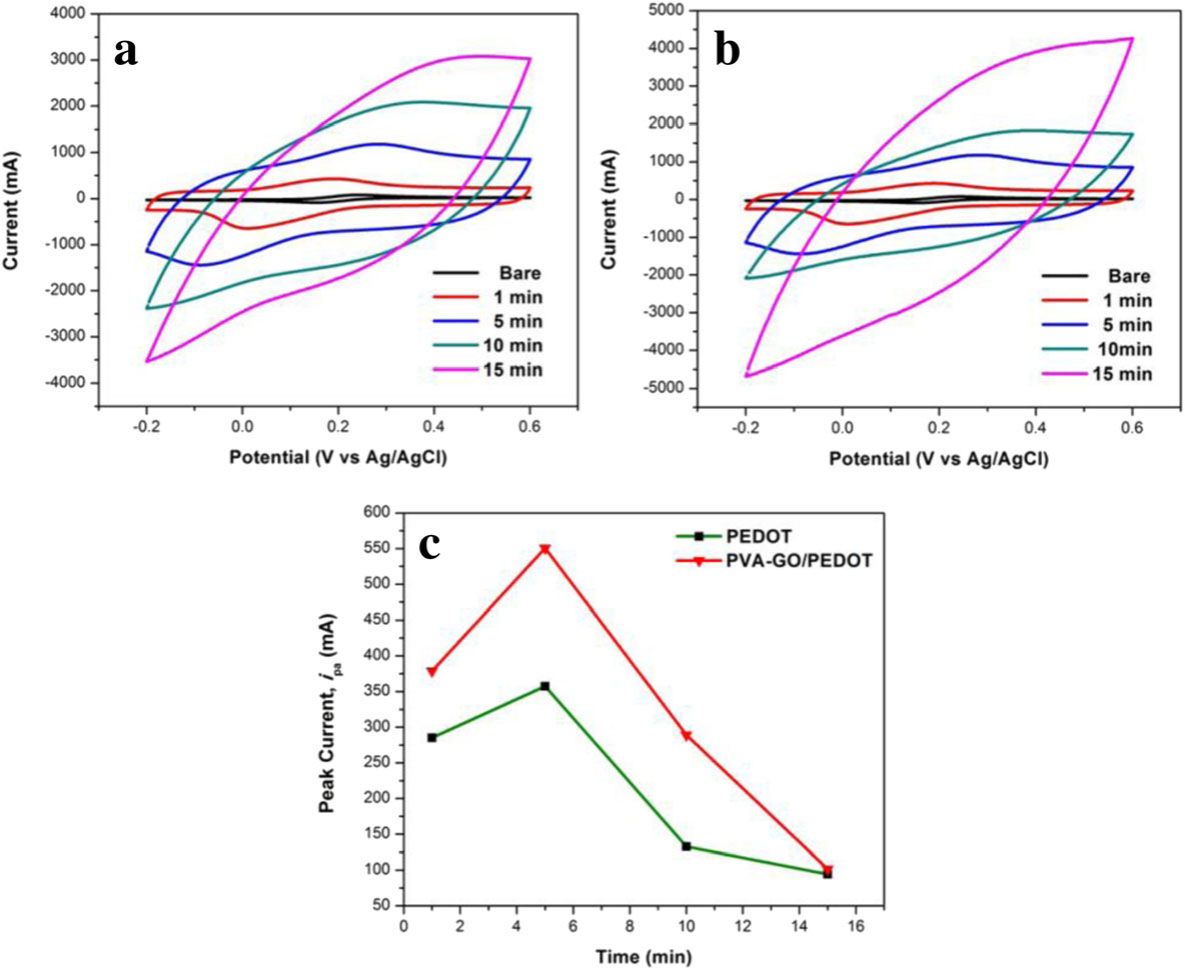
CVs of **a** PEDOT **b** PVA-GO/PEDOT prepared at different electropolymerization times of PEDOT in 1.0 mM K3Fe(CN)6 containing 0.1 M KCl solution. **c** Plot of anodic peak current at various electropolymerization times

In order to study the effect of electropolymerization time, PEDOT was electropolymerized at a constant potential (1.2 V) and varying the electropolymerization time from 1 to 15 min. By varying the deposition time, the physical properties such as the particles size, surface roughness, and thickness of PEDOT layer can be controlled to obtain a better electrochemical performance of coated nanofibers. The movement of ions to the electrode is expected to be easier if the thickness of the PEDOT layer is optimized which provides a high amount of active site area. As presented in Fig. 8a, as the electropolymerization time increase, the anodic peak becomes broader and the reduction peak is less observable. A similar result is noticed for PVA-GO/PEDOT nanofiber when the deposition time is extended to 15 min (Fig. 8b).

For PVA-GO/PEDOT1m nanofibers, the peak current is 378.75 mA. Subsequently, the value is further increased when the electropolymerization time increases to 5 min (550.59 mA). At this point, the peak current reached its maximum value. However, the peak current tends to decrease as the electrodeposition time is extended to 10 and 15 min. A Similar trend was observed for PEDOT deposited on bare ITO. The change in peak current can be ascribed to the difference in surface morphology of the PEDOT layer as the time increases. This might be attributed to the excessive overlapping and steric effect of the thicker PEDOT-coated layer when the polymerization time increases [38]. Therefore, the active surface area is blocked and consequently restricted the ion transportation at the electrode interface. This feature indicates clearly that the electropolymerization time of PEDOT is significantly changed the properties of PEDOT film.

At 15 min electrodeposition of PEDOT, the surface of PVA-GO/PEDOT15m nanofibers is fully covered with the compact PEDOT layer and partially hindered the electron transfer of redox probes to the active sites of nanofiber (Fig. 6f), thereby decreasing the current response. Additionally, the PVA-GO/PEDOT15m nanofibers give rise to a wide capacitive-like current response (Fig. 8b). A broad oxidation peak is seen between 0.25 and 0.40 V, but no clear reduction peak is observed during the reverse scan. It indicates that the transfer of electron to the electrode surface is limited and less accessible.

In contrast, the CV of PVA-GO/PEDOT5m displays the highest peak current response (Fig. 8c) which might be due to the fine coated of PEDOT layer on the nanofiber. The small grain of PEDOT-coated PVA-GO nanofibers contributes to the high surface area and large surface-to-volume ratio (Fig. 5b), which provides more active sites and short diffusion distances [39] resulting in low resistance for ion transfer processes. Thus, PVA-GO/PEDOT5m is considered the optimum time for the formation of PEDOT-coated layer.

### Impedance Analysis

**Fig. 9:**
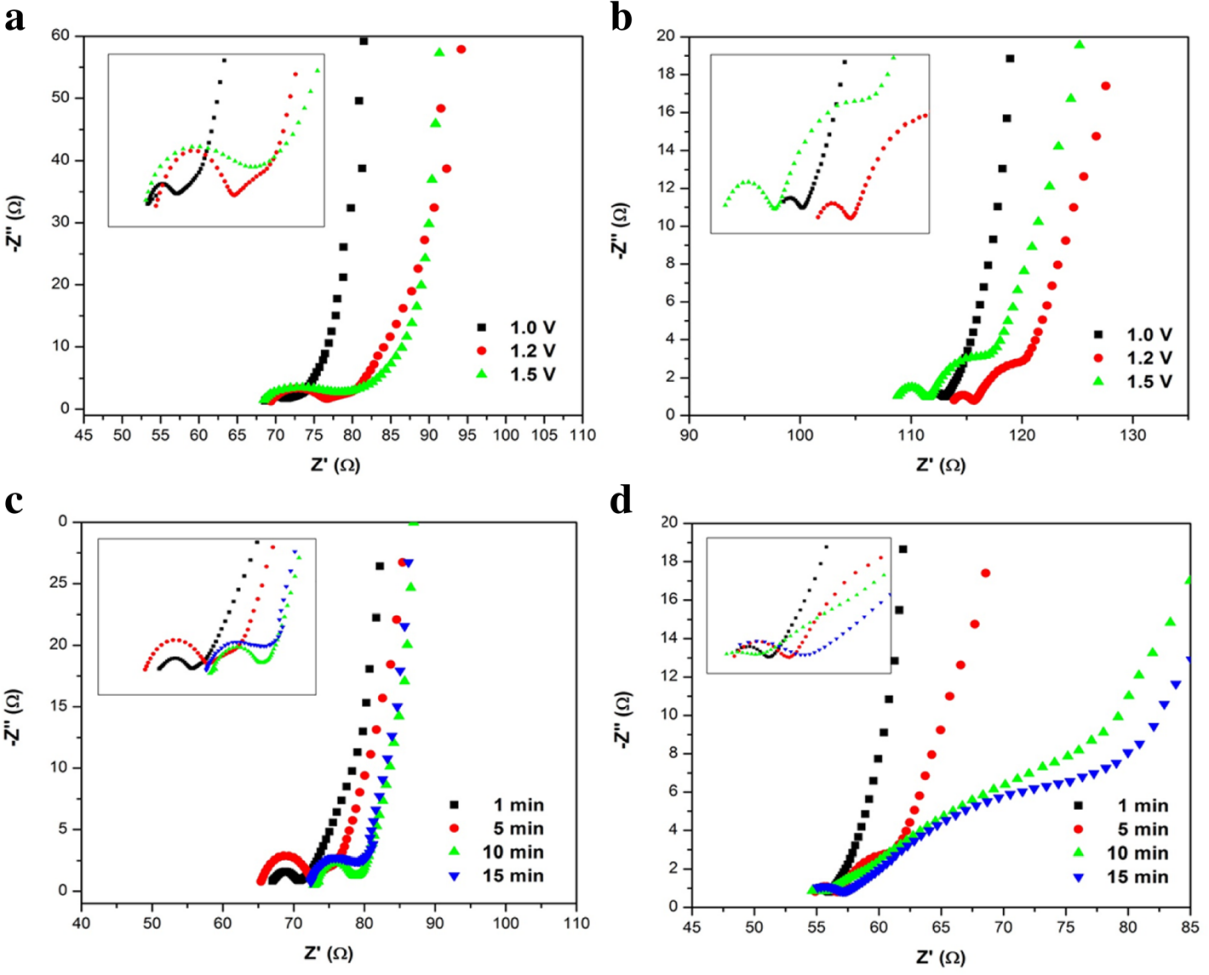
Nyquist plot of **a** PEDOT **b** PVA-GO/PEDOT at different electropolymerization potentials and **c** PEDOT **d** PVA-GO/PEDOT at different electropolymerization times of PEDOT in 5 mM [Fe(CN)6]^3−/4−^ and 0.1 M KCl. *Inset* Magnified representation of the Nyquist plot at the high-frequency region

The impedance of PEDOT and PVA-GO/PEDOT nanofibers prepared at different electropolymerization potentials of PEDOT was measured and the results are shown in Fig. 9a, b. All the Nyquist plots of PEDOT consist of a semicircle (Fig. 9a) followed by a linear behavior at the low-frequency region. In contrast, the Nyquist plots of PVA-GO/PEDOT1.2V and PVA-GO/PEDOT1.5V demonstrate of two semicircles at the high-frequency region (inset Fig. 9b, d) followed by a straight line slanted at a lower frequency. These two semicircles represent the bilayers of the two modified electrodes, which indicate two charge transfer processes with different time constants [40]. The left semicircle (*R*_ct1_) can be associated with the interfacial resistance between the PVA-GO nanofiber with the PEDOT-coated layer, followed by the second semicircle (*R*_ct2_) which arises from the charge transfer resistance between the PEDOT and the electrolyte solution in the electrochemical system. A linear line inclined at the lower frequency region is attributed to the semi-infinite diffusion of redox species from the bulk solution to the electrode surface. However, the Nyquist plot of PVA-GO/PEDOT1.0V (Fig. 9b) only reveals a single semicircle which later continues by a linear straight line. The second semicircle that represents the charge transfer resistance at the PEDOT|electrolyte interface that supposed to appear was not observed, could be due to the thin layer of PEDOT-coated nanofiber as observed in the SEM micrograph.

It was observed that the *R*_ct_ values for PVA-GO/PEDOT increase with the increasing of electrodeposition potential of PEDOT (Table 1). An increase in the *R*_ct1_ value indicates a higher resistance for the electron transfer between the PVA-GO nanofibers and the PEDOT layer due to the agglomerated and compact structure of the PEDOT-coated PVA-GO nanofiber (PVA-GO/PEDOT1.5V) due to the overoxidation during the electrodeposition [36], leading to the decrease of the conductivity of PEDOT polymer, and decelerates the electron transfer from the electrolyte to the electrode surface. A similar pattern was observed for PEDOT deposited on bare ITO which indicates augmentation of *R*_ct_ values as the electrodeposition potential increases (Table 1).

**Table 1:** Parameters obtained from electrochemical analysis at different electropolymerization conditions

Sample	Anodic peak current, *i*_pa_ (mA)	Resistance of charge transfer	
		*R*_ct1_ (Ω)	*R*_ct2_ (Ω)
PEDOT1.0V	156.90	–	3.21
PEDOT1.2V	357.24	–	8.85
PEDOT1.5V	223.69	–	11.96
PEDOT1m	285.37	–	5.19
PEDOT10m	132.65	–	8.32
PEDOT15m	94.29	–	9.26
PVA-GO/PEDOT1.0V	470.36	0.65	–
PVA-GO/PEDOT1.2V	550.59	1.44	6.32
PVA-GO/PEDOT1.5V	357.24	2.39	6.87
PVA-GO/PEDOT1m	378.75	1.37	–
PVA-GO/PEDOT10m	289.24	1.12	17.65
PVA-GO/PEDOT15m	101.47	2.59	17.81

Figure 9c, d shows the Nyquist plots of PEDOT and PVA-GO/PEDOT nanofibers prepared at different polymerization times of PEDOT. As can be seen in Fig. 9c (inset), PEDOT1m displays only a single semicircle, which is similar result was observed for PVA-GO/PEDOT1m (Fig. 9d). This phenomenon can be explained that the charge transfer resistance between the PVA-GO nanofibers and PEDOT, (*R*_ct1_), that supposed to appear at the high-frequency region might overlap with the semicircle that denoted by the resistance at PEDOT|electrolyte interface due to the small difference in their time constants. In contrast, PVA-GO/PEDOT5m, PVA-GO/PEDOT10m and PVA-GO/PEDOT15m show two semicircles in the Nyquist plots show two semicircles. The second semicircle, *R*_ct2_, at the lower frequency region exhibits a larger arc compared to *R*_ct1_ at the higher frequency region. By reviewing the diameter of these two semicircles (*R*_ct1_ and *R*_ct2_), it shows that the resistance increases at higher deposition time due to an increase in the thickness of the film during electropolymerization (Table 1). It was noticed that the difference in the electrodeposition time leads to different in the morphologies and the thickness of PEDOT-coated layer on nanofiber. For instance, PEDOT5m exhibited homogeneous and fibrous morphology with a smaller diameter of nanofiber compared to PEDOT10m and PEDOT15m. Thus, this nanofiber structures might create more active sites for electrical contact between polymer and redox species. These features might contribute to a higher active surface area that facilitates the ion transport process.

As expected, the formation of PEDOT on the PVA-GO nanofiber gives a significant impact on the charge transfer process at the PEDOT|electrolyte interface. The changes in the values of *R*_ct1_ and *R*_ct2_ were noticed as the electrodeposition time of PEDOT on PVA-GO nanofiber is increased to 15 min. A similar trend was also observed for PEDOT deposited on bare ITO. The increment of electrodeposition time leads to the formation of compact and thicker layer of PEDOT-coated PVA-GO nanofibers as can be seen in SEM images (Fig. 6). As a result, the resistances of the charge transfer could be related to the thickness of PEDOT-coated PVA-GO nanofibers, where in a thicker layer, the ion transport at the PEDOT|electrolyte interface is very poor, leading to slow the electron transfer at the electrode interface. Subsequently, the ions from the electrolyte have difficulty to diffuse into the surface of PEDOT layer.

**Fig. 10:**
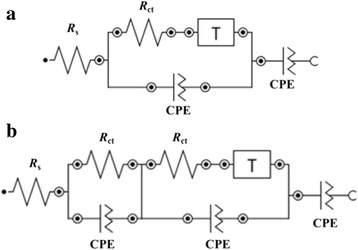
Equivalent electrical circuits for **a** PEDOT and **b** PVA-GO/PEDOT

The EIS data were fitted with equivalent electrical circuits (Fig. 10) in order to explain the behavior of the PEDOT and PEDOT-coated PVA-GO-modified electrodes. The accuracy of the fitted data is determined based on the chi-square (*χ* ^2^) which displays a small value (*χ* ^2^ = 10^−2^ to 10^−3^), indicating that the model is well fitted with the experimental data. The proposed models were constructed using four components. The first component is the *R*_s_, followed by the series combination of CPE and *R*_ct_. The equivalent circuits have a diffusion element (*T*) and the CPE as the last component, which corresponds to the capacitance element. *R*_s_ is the resistance of the bulk solution; constant phase element (CPE) is used to represent the non-ideal behavior of the double-layer capacitance and inhomogeneity of the electrode surface. Charge transfer resistance (*R*_ct_) is associated with the electron exchange at electrode–electrolyte interface, while the *T* element in the equivalent circuits refers to the ion diffusion which corresponds to a tangent hyperbolic function.

## Conclusions

PVA-GO/PEDOT-conducting nanofibers were successfully prepared by a combination of the electrospinning and electropolymerization methods. The concentration of GO in the polymer solution and the applied voltage during electrospinning process were optimized in order to obtain small diameter, uniform, bead-free, and continuous nanofibrous morphology. A proper coating of PEDOT layers on the surface of PVA-GO nanofibers was carried out at different electropolymerization parameters, and the optimized condition was 1.2 V of electrodeposition potential for 5 min. SEM images and structural analyzes revealed that the conducting polymer, PEDOT was effectively growth and well-coated on the electrospun nanofiber. PVA-GO/PEDOT nanofibers exhibited excellent electrochemical performance that can be used in various applications.

## Abbreviations

CNT: Carbon nanotubes
CV: Cyclic voltammetry
EIS: Electrochemical impedance spectroscopy
GO: Graphene oxide
ICPs: Intrinsically conducting polymers
ITP: Indium tin oxide
PEDOT: Poly(3,4-ethylenedioxythiophene)
PVA: Polyvinyl alcohol
PVA-GO: Polyvinyl alcohol-graphene oxide
SEM: Scanning electron microscope
